# Spectral attenuation curves of photon-counting detector CT scans for the differentiation between mucinous and non-mucinous cyst fluids of pancreatic lesions – a proof-of-concept study

**DOI:** 10.1186/s12880-025-01770-6

**Published:** 2025-07-01

**Authors:** Ibolyka Dudás, Borbála Lovász, Márton Benke, Máté Kiss, Markus Juergens, Ákos Szücs, Pál N. Kaposi, Attila Szijártó, Pál Maurovich-Horvat, Bettina K. Budai

**Affiliations:** 1 Medical Imaging Centre, Semmelweis University, Budapest, Hungary; 2 Department of Surgery, Transplantation and Gastroenterology, Semmelweis University, Budapest, Hungary; 3 Siemens Healthcare, Budapest, Hungary; 4 Siemens Healthineers AG, Computed Tomography, Forchheim, Germany; 5 Department of Diagnostic and Interventional Radiology, Heidelberg University Hospital, Heidelberg, Germany; 6 Medical Imaging Centre, Semmelweis University, 2 Korányi Sándor St, Budapest, 1083 Hungary

**Keywords:** Photon-counting detector, Computed tomography, Pancreatic cystic lesions, PCN, Spectral imaging

## Abstract

**Background:**

Spectral imaging via photon-counting detector CT (PCD-CT) scanners allows the generation of spectral attenuation curves. Our study aimed to test the feasibility of spectral attenuation curve analysis for differentiating mucinous from non-mucinous cyst fluids of pancreatic cystic lesions (PCL).

**Methods:**

Our study included 74 patients with PCLs. The classic morphological features were evaluated by an expert radiologist. The spectral attenuation curves were generated from the pancreatic-phase scans. The average CT values were measured on the 70 keV (HU_70keV_) and 40 keV (HU_40keV_) virtual monoenergetic images (VMIs), and the differences were calculated (HU_Δ40−70keV_). The cases were divided into training (53 mucinous vs. 19 non-mucinous PCLs) and test (10 mucinous vs. 10 non-mucinous PCLs) datasets. The receiver operating characteristic (ROC) curve analysis assessed the discrimination performance. The intra- and interobserver reproducibilities were evaluated by the intraclass correlation coefficient (ICC).

**Results:**

On 70 keV VMIs, no significant differences were found between the average CT values of mucinous and non-mucinous PCLs, however, the difference was significant in HU_Δ40−70keV_ values (*p* < 0.0001). The diagnostic performance of HU_Δ40−70keV_ in differentiating between mucinous vs. non-mucinous PCL had AUCs of 0.92 and 0.92 on the training and test datasets, respectively, with a good interobserver (ICC = 0.82) and excellent intraobserver reproducibility (ICC = 0.94). The conventional clinical-radiological model achieved AUCs of 0.85 and 0.86, and with the addition of HU_Δ40−70keV_ values its performance increased significantly to 0.98 and 0.94 on the training and test datasets, respectively.

**Conclusions:**

Spectral attenuation curve assessment of cyst fluids could be a useful additional measurement to facilitate the noninvasive differential diagnosis of PCLs.

**Supplementary Information:**

The online version contains supplementary material available at 10.1186/s12880-025-01770-6.

## Background

In recent decades, pancreatic cystic lesions (PCLs) have been increasingly identified as incidental findings in abdominal computed tomography (CT) scans, due to technological advancements in medical imaging and increased accessibility to imaging investigations [1, 2].

PCLs can be neoplastic or non-neoplastic, and pancreatic cystic neoplasms (PCN) can be further categorized into benign, premalignant, and malignant lesions according to the World Health Organization (WHO) classification system [3]. The most common PCLs include non-mucinous lesions such as pseudocysts (non-neoplastic), as well as neoplastic serous lesions such as serous cystic neoplasms (SCNs) and solid pseudopapillary neoplasms (SPNs), while the most frequent mucinous lesions are intraductal papillary mucinous neoplasms (IPMNs) and mucinous cystic neoplasms (MCNs). The malignant potential of mucinous and non-mucinous lesions shows marked differences; therefore, an accurate preoperative diagnosis has important prognostic and therapeutic implications and is essential for appropriate patient management [4, 5].

Although the classic morphologic features of IPMN, MCN, SCN, and SPN on CT scans are well described, in daily clinical practice, accurate differentiation remains challenging due to overlapping morphologies [6, 7]. Several studies reported poor accuracy in distinguishing between mucinous vs. non-mucinous PCLs based on visual assessment of CT scans alone [8–10]. When lesions show features suggesting malignancy on medical imaging examinations, endoscopic ultrasound (EUS) is recommended for confirmation [4, 5]. However, EUS-based diagnosis is subject to non-negligible interobserver variability [11], and although EUS-guided fine-needle aspiration (FNA) allows for biopsy/cytology and fluid analysis, diagnostic success remains limited. Previous studies reported that the success rate can be as low as 25% for EUS-FNA for PCLs [12], and the results can be inconclusive in approximately 50% of the cases [13]. A recent meta-analysis reported a pooled sensitivity of 63% and a specificity of 88% for EUS-based cytology and cyst fluid analysis in the differentiation of mucinous from non-mucinous PCLs [14].

Therefore, in recent years, there has been an urgent need for the development of noninvasive, imaging-based approaches to the early differentiation of mucinous and non-mucinous PCLs. Previous studies assessed the feasibility of utilizing dual-energy CT and dual-layer spectral CT scanners’ spectral information in pancreas imaging, including the assessment of CT values on virtual monoenergetic images (VMI). However, these previous studies mainly focused on solid tumors of the pancreas [15–18], and only few studies evaluating PCLs [10, 19, 20] can be found in the literature.

Mucinous cystic tumors of the pancreas have mucin-secreting cells, making the cyst fluid protein-rich, mucoid, and viscous, while serous cystic tumors and pseudocysts do not produce mucin, and their cyst fluid is thin and clear, with a more water-like consistency. Conventional CT scans are limited in evaluating cystic fluid content, as water and the different types of cyst fluids appear as similar hypodense lesions, also referred to as “fluid density” lesions (around 0–20 HU average CT density). Spectral imaging may overcome this limitation by analyzing voxel attenuation across different energy levels using VMI reconstructions.

In recent years, photon-counting detector CT (PCD-CT) scanners, which enable the acquisition of spectral information from the imaged structures, have been introduced to abdominal imaging [21]. A preliminary study by Laukamp et al. [22] reported that the use of 40 keV VMI reconstructions of PCD-CT scans significantly increased the diagnostic confidence of radiologists and the interobserver agreement of detecting small-sized PCLs compared to the reading of conventional CT series. However, to date, no studies have evaluated the use of spectral attenuation curves or keV-based attenuation differences from PCD-CT for differentiating PCL types.

In this study, we hypothesize that spectral imaging of PCD-CT can more precisely assess cyst fluid composition. Our study aimed to investigate whether the spectral attenuation characteristics, specifically the attenuation values of PCL cyst fluids at 40 keV and 70 keV as well as their difference, could help distinguish mucinous from non-mucinous PCLs. We also evaluated the intra- and inter-observer reproducibility of the proposed approach and demonstrated the added value of the spectral characteristic in comparison to the diagnostic performance of classical radiomorphological features.

## Methods

Our study was approved by the institutional ethics committee. As this was a retrospective study, the requirement for written informed patient consent was waived by the ethics committee. Clinical trial number: not applicable. All patient data were analyzed anonymously.

### Patient population

**Fig. 1:**
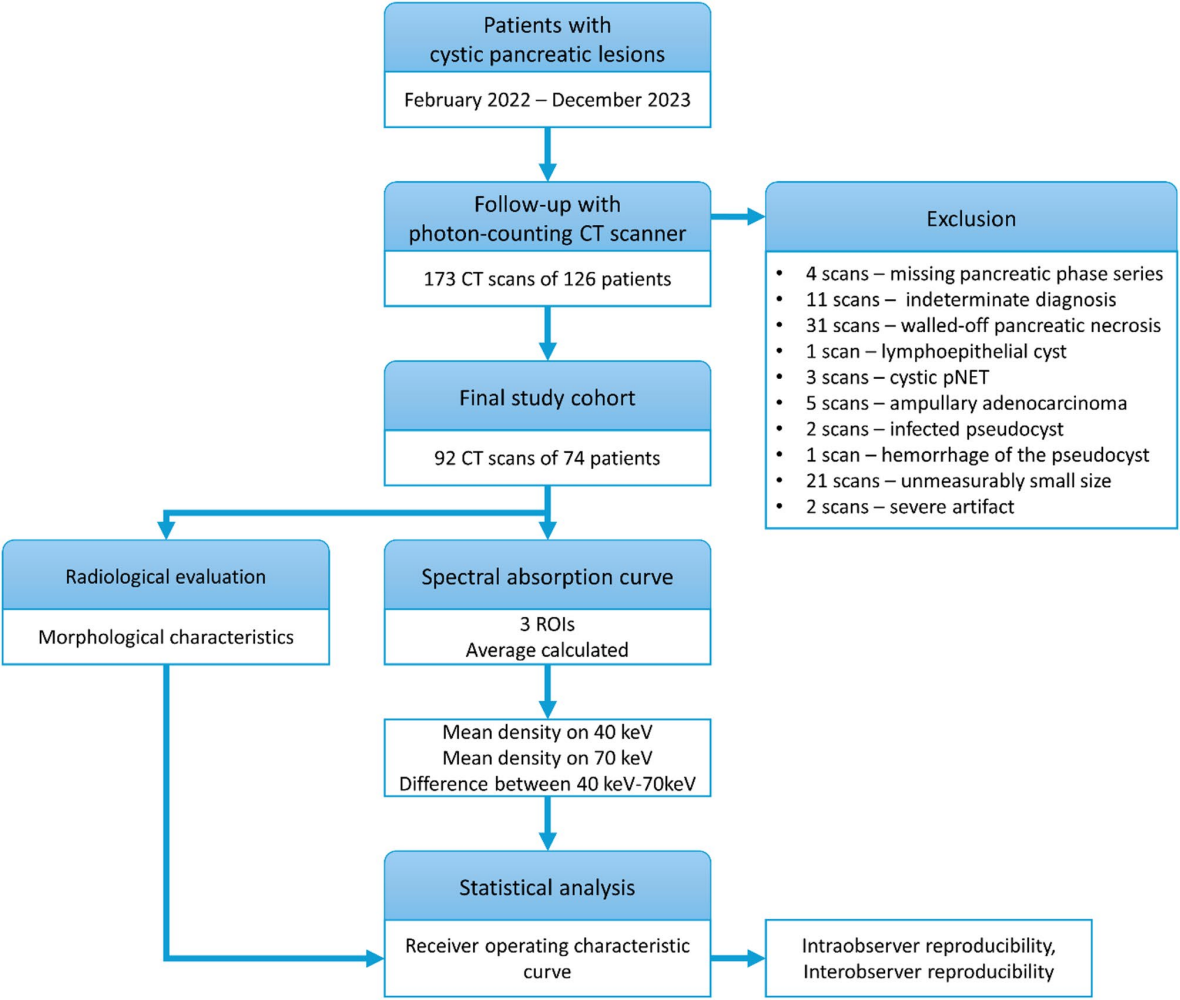
Flowchart on the study design and statistical analysis strategy. pNET: pancreatic neuroendocrine tumor; ROI: receiver operating characteristic curve

Consecutive patients who were diagnosed and followed for PCLs in our Surgery Department following the European evidence-based Guidelines of the European Study Group on Cystic Tumours of the Pancreas [5] and who were referred for follow-up CT examination at our Radiology Department between February 2022 and December 2023 were retrospectively enrolled. All consecutive patients who were diagnosed with PCLs were retrospectively identified which resulted in a total of 173 CT scans of 126 patients. Figure 1 shows the patient selection and data analysis steps. The exclusion criteria were the following: missing pancreatic phase series; indeterminate diagnosis; diagnosis of lymphoepithelial cyst, cystic pNET, ampullary adenocarcinoma, walled-off pancreatic necrosis; hemorrhage or infected pseudocyst; unmeasurably small size; artifact affecting image quality. The minimum size limit of the lesion was defined as internal cystic fluid measurable with a region of interest (ROI) of at least 0.2 cm^2^. The final study cohort included 74 patients with 92 CT scans.

The etiology of the PCLs was confirmed by a combination of clinical and pathological criteria documented in the hospital information system. Pathological confirmation of the neoplasms was available for 24 patients, including 14 cases proved by EUS-FNA biopsy/cytology, and 10 cases with available final histopathology after surgical resection. The diagnoses of the 9 patients with pseudocysts were obtained based on the consensus of medical history, patient presentation, and CT imaging findings. For the remaining 41 neoplastic cases, the clinically presumed pathology was determined by the consensus opinion of the institutional multidisciplinary tumor board based on medical history, previous follow-up results, and EUS and/or MRI/MRCP examination results. While this inclusion reflects real-world clinical practice and expert evaluation, the absence of histological confirmation in a subset of cases may have introduced bias or affected the robustness of the model’s performance metrics. Given that this study is intended as a proof-of-concept study, these limitations should be considered when interpreting the results.

### Imaging protocol

The patients were examined at our institution with a PCD-CT scanner (NAEOTOM Alpha, VA50; Siemens Healthineers) according to our routine pancreas imaging protocol. For the postcontrast phase series, a nonionic iodinated contrast agent (either Ultravist 370 or Iomeron 350; amount adjusted to the patient’s body weight) was injected at an injection rate of 2.4–4.3 mL/sec. The timing for the pancreatic phase series was 45 s. The scans were performed with a tube voltage of 120 kVp, an automated tube current modulation, a rotation time of 0.5 s, a pitch of 0.80, a total collimation width of 144 × 0.4 mm, and a 512 × 512 reconstruction matrix. Axial reconstructions were generated with the SPP spectral reconstruction algorithm using a Qr40 soft kernel and a Q3 quantum iterative reconstruction algorithm with a slice thickness of 2.0 mm. The detailed imaging protocol can be found in Supplementary Material (see Additional file 1).

### Spectral attenuation curve assessment

**Fig. 2:**
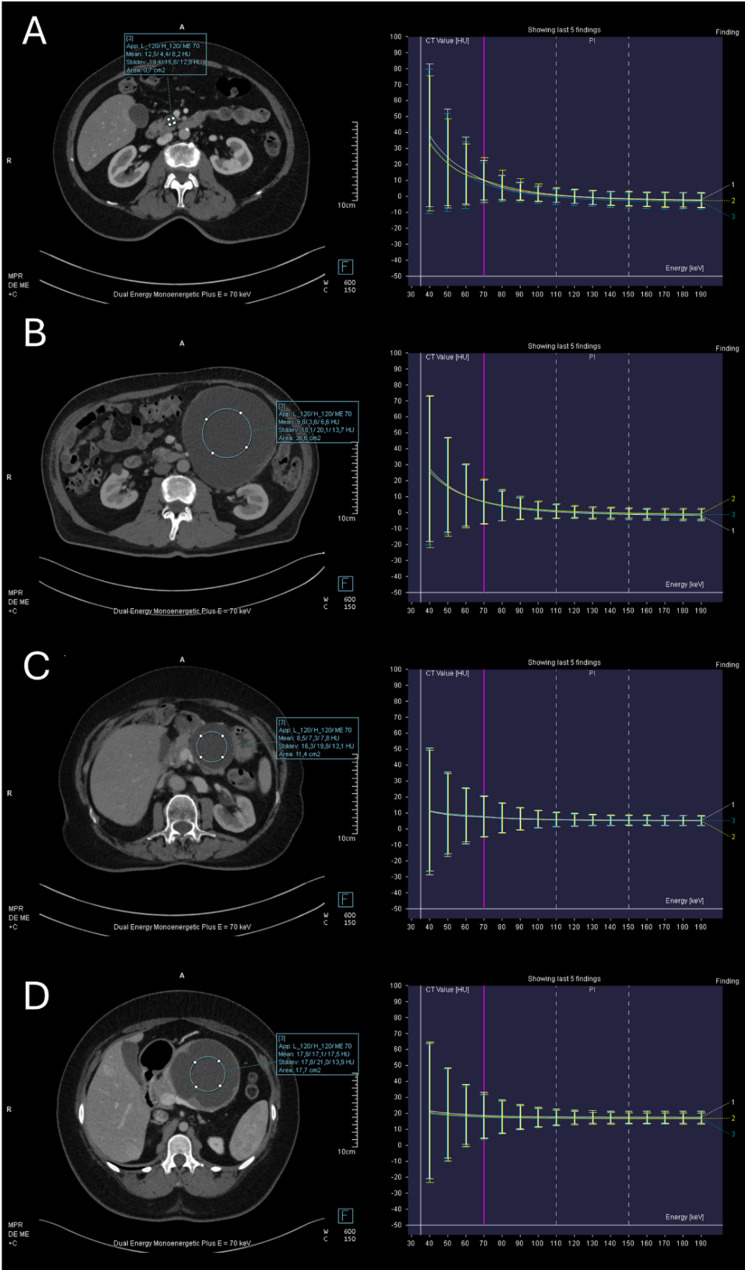
Spectral attenuation curves of mucinous and non-mucinous pancreatic lesions. Intraductal papillary mucinous neoplasm (**A**), Mucinous cystic neoplasm (**B**), pseudocyst (**C**), and cystic dominant solid pseudopapillary neoplasm (**D**) on 70 keV virtual monoenergetic images and the spectral attenuation curves of cyst fluids

The quantitative assessment of the PCLs was performed on the pancreatic phase series by using the Monoenergetic + application of the Dual Energy module (syngo.via software, VB60; Siemens Healthineers). Three circular ROIs were manually placed in cyst fluids on the VMI reconstructions while taking care to avoid internal septa, mural nodules, calcifications, and cyst walls. The spectral attenuation curves were plotted on a graph as Hounsfield Unit (HU) values against different keV VMI reconstructions (Fig. 2). The CT values on the 40 keV VMIs (HU_40keV_), and the 70 keV VMIs (HU_70keV_), as well as the difference between 40 keV and 70 keV (HU_Δ40−70keV_) were extracted. The average of the three measurements was calculated for each case. Water in the stomach was also measured for comparison by placing a single ROI. Five patients refused to drink water; therefore, spectral characteristics of the water were only measurable in 87 CT scans.

### Reproducibility assessment

The spectral CT value measurements were carried out by an expert radiologist specializing in abdominal imaging with 15 years of experience who was blinded to the patients’ medical history and diagnosis. For intraobserver reproducibility assessment, the same radiologist repeated the measurements 4 months later blinded to the previous measurement results. For interobserver reliability, a trainee with 4 years of experience in abdominal imaging also carried out the measurements blinded to the patients’ medical history, their diagnosis, and the measurements of the expert radiologist.

### Morphological evaluation

The radiological evaluation of the PCLs was conducted by the same expert radiologist. The pancreatic lesions were evaluated by reading the 40 keV and 70 keV VMI reconstructions of the pancreatic phase CT series. The assessed morphological features included the contour (smooth/lobulated/irregular), cyst structure (monolocular/oligolocular/multilocular), Wirsung duct dilatation (no/<5 mm/5–9 mm/>10 mm), lesion communicates with the main pancreatic duct (yes/no), lesion communicates with the branch ducts (yes/no), larger diameter (mm), cyst diameter (< 30 mm/>30 mm), solid component (yes/no), internal septa (yes/no), thickened or enhancing septa (yes/no), enhancing mural nodule (no/<5 mm/>5 mm), thickened/enhancing cyst wall (yes/no), abrupt change in caliber of the pancreatic duct with distal pancreatic atrophy (yes/no), mural calcification (yes/no), central calcification (yes/no), pancreas parenchyma (normal/atrophy/chronic pancreatitis/fatty transformation/ not assessable).

### Diagnostic performance assessment

To assess the diagnostic performance of the clinical-radiological features and the extracted spectral CT values, the cases were randomly divided into training and test datasets. The test data was compiled by selecting 10 mucinous and 10 non-mucinous PCL cases using a random generator. The training set with 53 mucinous lesions and 19 non-mucinous lesions was used for constructing logistic regression models, and the models’ performances were tested on both the training and the test cases.

### Statistical analysis

For statistical analysis, the Shapiro-Wilk test was used to assess normality. Fisher’s exact test and two-tailed Mann-Whitney U test were used to compare the categorical and continuous variables. The differences in CT values between the fluid groups were evaluated by using the Kruskal-Wallis test with the post-hoc Dunn’s test. Correction for multiple testing was applied according to the Benjamini-Hochberg method. A threshold of *p* < 0.05 was used to determine the statistical significance. Logistic regression analysis was used to identify morphological features associated with lesion type. The variables with *p* < 0.1 in the univariate analysis were entered into the multivariate logistic regression analysis. The performance of the quantitative measurements as well as the radio-morphological features in differentiating between mucinous vs. non-mucinous PCLs was evaluated based on receiver operating characteristics (ROC) curve analysis. McNemar’s test was used to directly compare the models’ performances.

Intraclass correlation coefficients (ICCs) and 95% confidence intervals (CIs) were used to evaluate the intra- and interobserver reproducibility using two-way random effects, single measurement, and absolute agreement models. The interobserver agreement was evaluated based on Gwet’s AC1 coefficient values, and Bland-Altman plots. The agreement based on Gwet’s AC1 coefficient values was classified as 0.8–1.0 (very good), 0.6–0.8 (good), 0.4–0.6 (moderate), 0.2–0.4 (fair), and < 0.2 (poor).

The statistical analysis was conducted with dedicated packages coded in R and Python.

## Results

### Patient population

The final patient cohort included 1 patient (1 CT scan) with mucin-producing adenocarcinoma, 46 patients (54 CT scans) with IPMNs, 6 patients (8 CT scans) with MCNs, 9 patients (11 CT scans) with pseudocysts, 11 patients (17 CT scans) with SCNs, and 1 patient (1 CT scan) with a cystic dominant SPN. For further analysis, the patients were categorized into two groups: mucinous PCLs (53 patients, 63 CT scans), and non-mucinous PCLs (21 patients, 29 CT scans). Pathological confirmation was available for 17 patients with mucinous and 3 with non-mucinous PCLs. The distribution of age and the male-to-female ratio were similar between the two groups, with 67.4 ± 12.6 vs. 61.1 ± 16.9 years (*p* = 0.2214); and 15 males (28.3%) vs. 6 males (28.6%) (*p* = 1.0), respectively.

### Morphological assessment of the lesions

Morphological evaluation of the PCLs revealed significant differences between mucinous and non-mucinous PCLs in terms of contour (*p* = 0.0476), communication with the pancreatic ducts (*p* < 0.0001), largest diameter as a continuous variable (*p* = 0.0001), and cyst size larger than 30 mm as a binary variable (*p* = 0.0033). Compared with those in the mucinous group; the non-mucinous lesions had a significantly larger diameter, moreover, we found that most of the lesions had a size of > 30 mm in the non-mucinous group, while in the mucinous group, most of the lesions had a size of < 30 mm. The tumor contour was mainly lobulated in both lesion types, however, the proportion of lesions with lobulated contours was higher (16/29 vs. 46/63) and the proportion of those with irregular borders was lower (5/29 vs. 2/63) in the mucinous group. As expected, none but one lesion showed communication with the pancreatic duct in the non-mucinous group, while in the mucinous group, 35/63 and 53/63 lesions showed communication with the main pancreatic duct or branch ducts, respectively. The morphological characteristics are summarized in Table 1.

**Table 1:** Morphological characteristics of the pancreatic cystic lesions

Characteristics	Non-mucinous lesions	Mucinous lesions	*p*-value
number of CT scans	29	63	-
sex (female/male)	21 / 8	46 / 17	1.0000
age (years)*	62.52 ± 15.95	67.00 ± 13.00	0.2214
contour (smooth/lobulated/irregular)	8 / 16 / 5	15 / 46 / 2	**0.0476**
cyst structure (monolocular/oligolocular/multilocular)	11 / 6 / 12	20 / 24 / 19	0.2478
Wirsung duct dilatation (no, <5 mm, 5–9 mm, > 10 mm)	16 / 11 / 2 / 0	31 / 18 / 6 / 8	0.1984
lesion communicates with the main pancreatic duct (yes/no)	1 / 28	35 / 28	**< 0.0001**
lesion communicates with the branch ducts (yes/no)	1 / 28	53 / 10	**< 0.0001**
larger diameter (mm)*	51.00 ± 42.93	29.67 ± 20.34	**0.0016**
cyst diameter (< 30 mm; >30 mm)	8 / 21	39 / 24	**0.0033**
solid component (yes/no)	1 / 28	5 / 58	0.6608
internal septa (yes/no)	19 / 10	44 / 19	0.8096
thickened or enhancing septa (yes/no)	0 / 29	3 / 60	0.5490
enhancing mural nodule (no/<5 mm/>5 mm)	28 / 1 / 0	57 / 2 / 4	0.5576
thickened/enhancing cyst wall (yes/no)	3 / 26	4 / 59	0.6743
abrupt change in caliber of the pancreatic duct with distal pancreatic atrophy (yes/no)	4 / 25	10 / 53	1.0000
mural calcification (yes/no)	2 / 27	3 / 60	0.6487
central calcification (yes/no)	2 / 27	1 / 62	0.2328
pancreas parenchyma (normal/atrophy/chronic pancreatitis/fatty transformation/ not assessable)	9 / 1 / 13 / 2 / 4	32 / 4 / 15 / 1 / 11	0.1336

* values expressed as mean ± standard deviation

### Comparison of the mean CT values

**Fig. 3:**
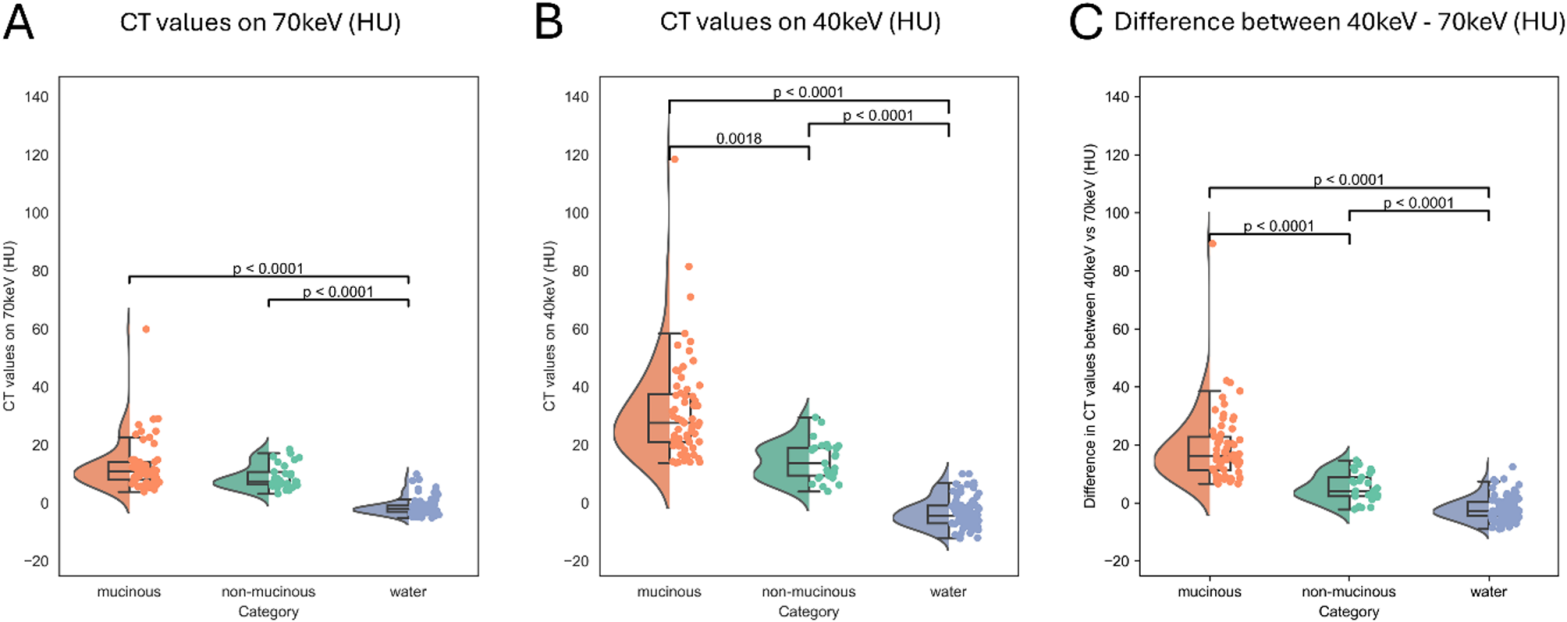
Boxplots on the differences in CT values between mucinous pancreatic lesions, non-mucinous pancreatic lesions, and water. No significant differences were found between lesions on the 70 keV virtual monoenergetic reconstructions (**A**). The mucinous pancreatic lesions showed significant differences from the non-mucinous pancreatic lesions at 40 keV (**B**). The differences between the lesions were most prominent in the case of the calculated difference between 40 keV and 70 keV CT values (**C**)

The HU_70keV_ values showed no significant differences between mucinous PCLs and non-mucinous PCL, however, both types of fluids showed significantly higher values than water (*p* < 0.0001). There was a significant difference in HU_40keV_ CT values between mucinous vs. non-mucinous PCLs (*p* = 0.0018), and between both fluids compared to water (*p* < 0.0001). In the case of HU_Δ40−70keV_ CT values, more significant differences were observed between mucinous vs. non-mucinous PCLs (*p* < 0.0001) (Fig. 3), which further confirms the difference in spectral attenuation pattern between mucinous and non-mucinous fluids.

### Diagnostic performance assessment

Univariate logistic regression analysis of the training dataset revealed that sex (*p* = 0.0959), age (*p* < 0.0001), lesion contour (*p* = 0.0092), cyst structure (*p* = 0.0016), main pancreatic duct dilatation (*p* = 0.0028), communication with the main pancreatic duct (*p* = 0.0009), presence of internal septa (*p* = 0.0003), and morphology of the pancreas parenchyma (*p* = 0.0610) showed association with lesion type (Table 2).

**Table 2:** Univariate logistic regression analysis of clinical-radiological parameters

Variable	OR	*p*-value
sex	2.14 [0.87–5.26]	**0.0959**
age	1.02 [1.01–1.03]	**< 0.0001**
contour (smooth/lobulated/irregular)	1.96 [1.18–3.27]	**0.0092**
cyst structure (unilocular/oligolocular/multilocular)	2.05 [1.31–3.2]	**0.0016**
Wirsung duct dilatation (no, <5 mm, 5–9 mm, > 10 mm)	2.64 [1.4–5]	**0.0028**
lesion communicates with the main pancreatic duct (yes/no)	29.00 [3.95–212.89]	**0.0009**
lesion communicates with the branch ducts (yes/no)	1.25E + 09 [0 - inf]	0.9968
larger diameter	1.00 [0.99–1.01]	0.4146
cyst diameter (< 30 mm; >30 mm)	1.29 [0.64–2.59]	0.4807
solid component (yes/no)	3.00 [0.31–28.84]	0.3414
internal septa (yes/no)	3.45 [1.77–6.76]	**0.0003**
thickened or enhancing septa (yes/no)	2.25E + 13 [0 - inf]	0.9999
enhancing mural nodule (yes/no)	3.22 [0.59–17.73]	0.1783
thickened/enhancing cyst wall (yes/no)	0.67 [0.11–3.99]	0.6569
abrupt change in caliber of the pancreatic duct with distal pancreatic atrophy (yes/no)	2.67 [0.71–10.05]	0.1474
mural calcification (yes/no)	1.50 [0.25–8.98]	0.6569
central calcification (yes/no)	2.25E + 13 [0 - inf]	0.9999
pancreas parenchyma (normal/atrophy/chronic pancreatitis /fatty transformation/ not assessable)	1.26 [0.99–1.6]	**0.0610**

CI: confidence interval; OR: odds ratio

**Fig. 4:**
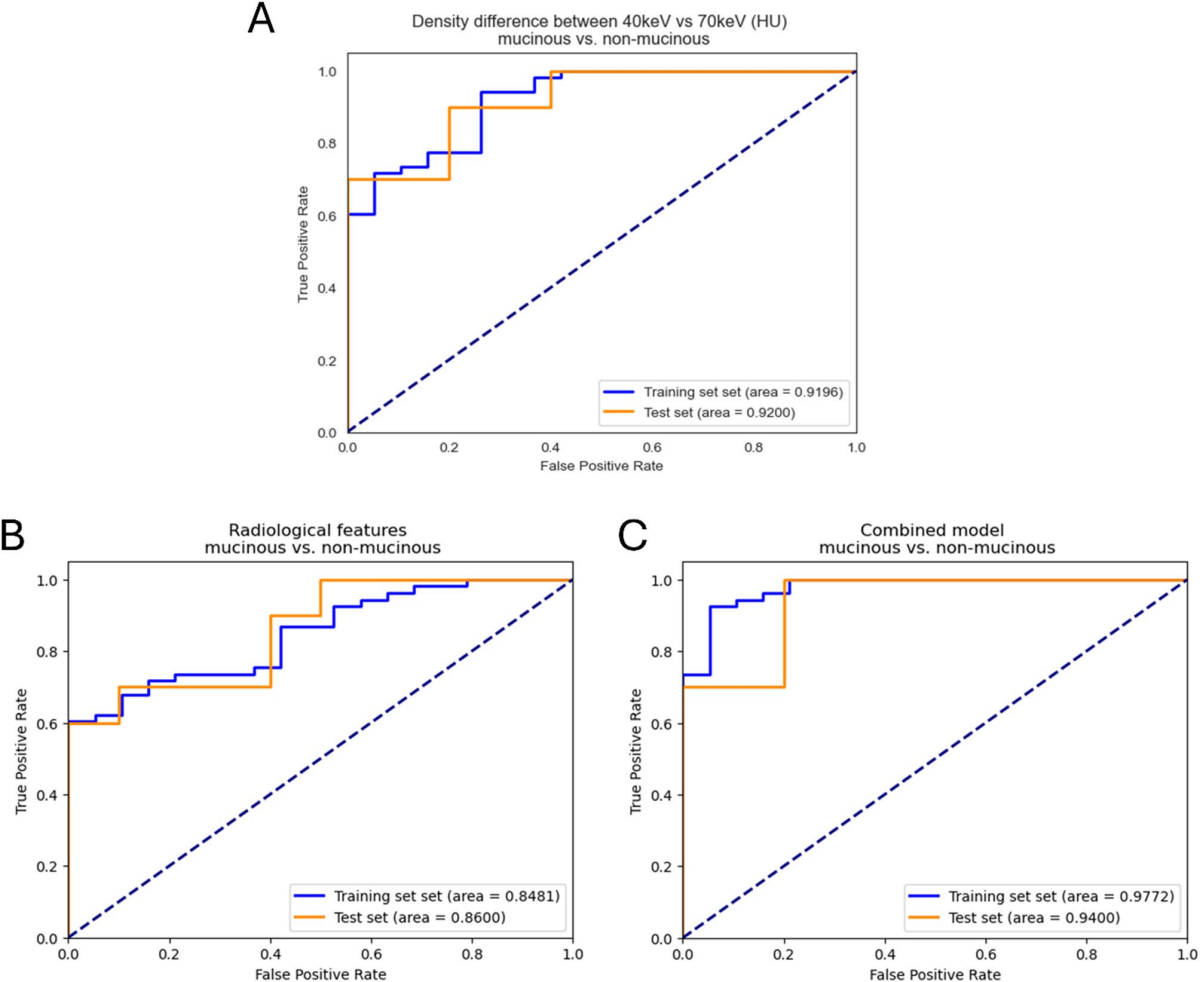
Receiver operating characteristic curve analysis for distinguishing between mucinous and non-mucinous cystic pancreatic lesions. The difference in CT values between 40 keV and 70 keV achieved similarly excellent discrimination ability on the training and test datasets (**A**). The conventional radiological model had slightly lower performance (**B**). Adding the spectral characteristic to the conventional features further increased its accuracy (**C**)

These eight promising variables were then used to construct the clinical-radiological logistic regression classification model. The model achieved a good diagnostic performance in differentiating between mucinous vs. non-mucinous lesions in the training dataset with an area under the ROC curve (AUC) of 0.85, an accuracy of 79.2%, a sensitivity of 96.2%, and a specificity of 31.6%, however, its performance on the test dataset was limited in terms of threshold-based metrics with an accuracy of 55.0%, a sensitivity of 100.0% and a specificity of 10.0% despite a comparable AUC of 0.86 (Fig. 4; Table 3).

**Table 3:** The diagnostic performance of the models on the training and test datasets

Model	Dataset	AUC	Accuracy	Sensitivity	Specificity	PPV	NPV	MCC
Combined model	Training set	0.9772	93.06%	96.23%	84.21%	94.44%	88.89%	0.8187
	Test set	0.9400	85.00%	100.00%	70.00%	76.92%	100.00%	0.7338
Clinical-radiological model	Training set	0.8481	79.17%	96.23%	31.58%	79.69%	75.00%	0.3899
	Test set	0.8600	55.00%	100.00%	10.00%	52.63%	100.00%	0.2294
HU_Δ40−70 keV_	Training set	0.9196	86.11%	94.34%	63.16%	87.72%	80.00%	0.6240
	Test set	0.9200	80.00%	90.00%	70.00%	75.00%	87.50%	0.6124
HU_40keV_	Training set	0.8739	80.56%	88.68%	57.89%	85.45%	64.71%	0.4833
	Test set	0.9000	70.00%	100.00%	40.00%	62.50%	100.00%	0.5000
HU_70keV_	Training set	0.6569	73.61%	100.00%	00.00%	73.61%	-	0.0000
	Test set	0.6800	50.00%	100.00%	00.00%	50.00%	-	0.0000

AUC: area under the receiver operating characteristic curve; HU: Hounsfield unit; PPV: positive predictive value; NPV: negative predictive value; MCC: Matthew’s correlation coefficient

Among the spectral characteristics, the HU_70keV_ values had no potential for discriminating between the mucinous vs. non-mucinous pancreatic lesions, while the HU_40keV_ values had good-to-excellent diagnostic potential with AUCs of 0.87 and 0.90, accuracies of 80.6% and 70.0%, sensitivities of 88.7% and 100.0%, and specificities of 57.9% and 40.0% on the training and test datasets, respectively. The HU_Δ40−70keV_ values yielded the best results with AUCs of 0.92 and 0.92, accuracies of 86.1% and 80.0%, sensitivities of 94.3% and 90.0%, and specificities of 63.2% and 70.0%.

Moreover, adding the HU_Δ40−70keV_ values to the clinical-radiological model significantly increased its diagnostic performance by increasing its specificity on both the training (*p* = 0.021) and test datasets (*p* = 0.031). The integrated model had AUCs of 0.98 and 0.94, accuracies of 93.1% and 85.0%, sensitivities of 96.2% and 100.0%, and specificities of 84.2% and 70.0% on the training and test datasets, respectively.

### Reproducibility assessment

**Fig. 5:**
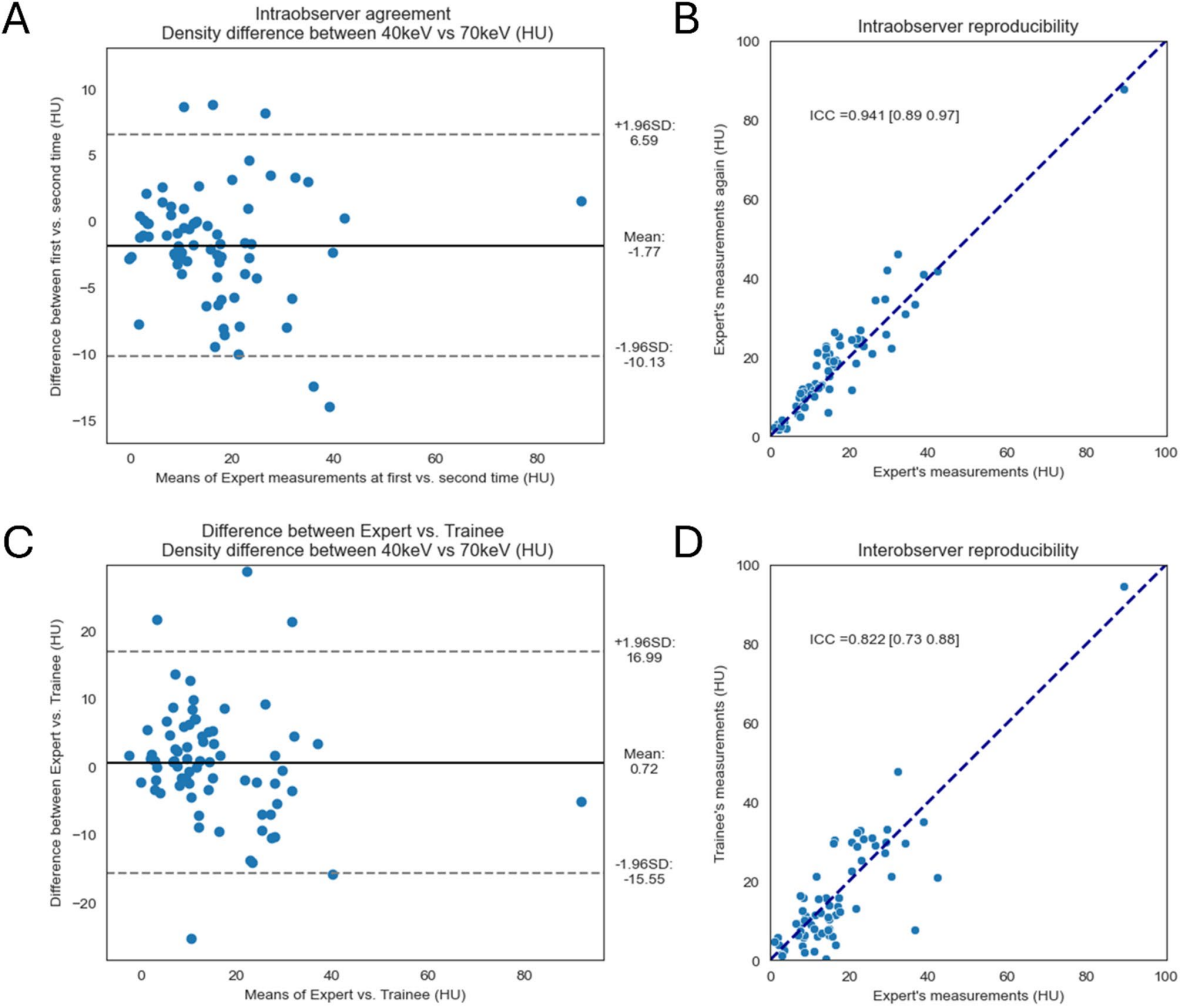
Intraobserver and interobserver reliability analysis. The intraobserver reproducibility analysis showed excellent (**B**), while the interobserver reliability showed good reproducibility (**D**). The Bland-Altman plot confirmed these results (**A**, **C**)

Our study also investigated the intra- and interobserver reproducibility of the measured HU_Δ40−70keV_ values. The interobserver reproducibility between an expert radiologist and a trainee assessed in the training dataset resulted in good reproducibility with an ICC of 0.82 [95% CI: 0.73–0.88]. The good reproducibility was also confirmed by Gwet’s AC1 coefficient value of 0.60 [95% CI: 0.41–0.79]. To assess the intraobserver reproducibility, the same expert radiologist repeated the spectral characteristics measurements after 4 months. The reliability analysis revealed excellent intraobserver reproducibility with an ICC of 0.94 [95% CI: 0.89–0.97] (Fig. 5). In both cases, the Bland-Altman plots showed that the measurements were distributed around the mean difference close to zero. Almost all the cases were within ± 1.96 SD. The difference between the two measures showed no dependency on the two measurements’ average values (Fig. 5).

## Discussion

In our study, we analyzed pancreatic phase abdominal PCD-CT scans of 74 patients with PCLs. The clinical-radiological model achieved only a limited performance in distinguishing between mucinous vs. non-mucinous PCLs with AUCs of 0.85 and 0.86 on the training and test datasets, respectively. During the assessment of the spectral attenuation curves, we measured the mean HU CT values of the cyst fluids on the 40 keV and 70 keV VMIs and calculated the difference between the two. The HU_70keV_ values showed no significant difference between mucinous vs. non-mucinous PCLs, the HU_40keV_ values had good discrimination ability with AUCs of 0.87 and 0.90, while the HU_Δ40−70keV_ values achieved the best results with AUCs of 0.92 and 0.92, which also showed excellent intraobserver (ICC = 0.94) and good interobserver (ICC = 0.82) reproducibility. Moreover, adding the HU_Δ40−70keV_ values to the clinical-radiological features significantly increased the performance, and the integrated model achieved similarly excellent results on the training and test datasets with AUCs of 0.98 and 0.94, respectively.

Previous studies that assessed the role of spectral information in the differentiation between mucinous and non-mucinous PCLs evaluated the spectral information generated by a fast kilovoltage-switching dual-energy CT scanner [10, 19, 20]. To the best of our knowledge, our study is the first to investigate this clinical question using a photon-counting CT scanner.

Lin et al. [10] were the first to evaluate the diagnostic value of spectral characteristics in distinguishing between SCNs and MCNs using fast tube-voltage switching dual-energy CT. Based on late arterial phase scans of 44 patients, only tumor location, contour, and diameter showed significant differences between MCNs and SCNs among the morphological features, with AUCs of 0.733–0.857. In our study, univariate logistic regression analysis revealed significant associations between PCL type and patient sex, patient age, cyst contour, cyst structure, main pancreatic duct dilatation, communication with the main pancreatic duct, presence of internal septa, and morphology of the pancreatic parenchyma. Lin et al. reported that the logistic regression model constructed from three morphological features yielded an AUC of 0.934, while in our study, we obtained slightly lower results on the training dataset with an AUC of 0.85 and an accuracy of 79%. This prior work demonstrated that lower keV VMIs (40–60 keV) enhanced the contrast between lesion types and that CT density values at lower energy levels had higher discrimination ability for serous vs. mucinous cystic neoplasms. During the spectral attenuation curve analysis, similar to Lin et al., we found significantly different HU_40keV_ CT values between the two lesion types but with better prediction performance (AUC of 0.707 vs. 0.874). Our findings align with theirs in showing that 40 keV provides better diagnostic performance than 70 keV, although our study could not confirm the significant differences in HU_70keV_ values or their diagnostic value. To extend these findings, our study also investigated the 40–70 keV attenuation difference as a novel feature, which yielded even better performance than either single energy level alone.

In the next study of the same group, Li et al. [19] built two support vector machine classifiers to distinguish between MCNs and SCNs, one from the clinical and morphological features, and one that also integrated the spectral characteristics. In this prior study, adding spectral features to their model increased its accuracy from 88.37 to 93.02%. The added value of spectral features to the clinical-radiological model was also confirmed in our study; the accuracy of our integrated model on the training dataset was equal to that reported by Li et al. (93.06% vs. 93.02%). However, our clinical-radiological model yielded limited performance on the test cases with an accuracy of 55.00% (vs. 79.17%), besides comparable AUC, which underlines the importance of reporting the results on independent test datasets.

Zhang et al. [20] investigated the value of spectral characteristics in differentiating between SPNs, MCNs, and pseudocysts by analyzing CT scans from 56 patients. Although this study was published by a different institution than the previous two articles discussed above, the scans were performed on the same fast kilovoltage-switching dual-energy CT model of the same vendor. In this study, the slope of the spectral attenuation curve was calculated, while in our study, to simplify the calculation and make the method easier to use in practice, we estimated the rise of the exponential curve approximately by calculating the difference between the CT values measured at 40 keV and 70 keV. Zhang et al. reported that the slope on portal venous phase scans was the highest for SPNs, followed by MCNs, while the lowest values were found for pseudocysts. In our study, we found similar attenuation curve patterns; the mucinous PCLs, including the MCNs and IPMNs, showed steeper curves compared to the non-mucinous lesions, including SCNs and pseudocysts, as demonstrated in Fig. 2. In this prior study, the spectral characteristics surpassed the performance of the morphological features in differentiating MCNs from pseudocysts, the slope had an AUC of 0.885 with a sensitivity of 90.9% and a specificity of 88.9%. These results are comparable to ours; however, in our study, we also included IPMNs in addition to MCNs in the mucinous group and SCNs, as well as pseudocysts and a cystic SPN in the non-mucinous group.

Although two of these previous studies built logistic regression-based models and one constructed a support vector machine algorithm for machine learning models, none of these studies evaluated their models’ performance on independent test cases. An important methodological advancement of our study is that we successfully demonstrated the added diagnostic value of the spectral characteristics to the clinical-radiological features by testing the model’s performance on an independent test dataset, which underlines the importance of quantitative spectral features. Moreover, we also investigated the intraobserver and interobserver reproducibility of the calculated HU_Δ40−70keV_ values, which demonstrated the good-to-excellent reliability of the proposed approach.

Our study has several limitations that need to be addressed. First, this was a single-center study with a retrospective study design. Our study included a relatively low number of patients; however, our patient cohort was larger than the three studies previously published in the literature (74 vs. 42, 44, and 56 patients). Histopathological diagnosis was not available for all cases, and for patients for whom CT scans did not show any worrisome features and EUS-FNA was therefore not warranted, the diagnosis was made based on the decision of the multidisciplinary tumor board.

## Conclusions

In conclusion, our study demonstrated the additional value of spectral characteristics compared to clinical-radiological parameters in differentiating between mucinous and non-mucinous cyst fluids of PCLs. We proved that the proposed quantitative approach has excellent intraobserver and good interobserver reproducibility. Our results showed that the assessment of spectral characteristics of PCD-CT scans can provide valuable quantitative information and could be a promising additional non-invasive tool for the differentiation of mucinous from non-mucinous PCLs.

## Electronic supplementary material

Below is the link to the electronic supplementary material.

Supplementary Material 1

## Data Availability

The CT images analyzed during the current study are not publicly accessible due to Hungarian medical data privacy guidelines. The anonymized tabular data used and/or analyzed during the current study are available from the corresponding author upon reasonable request.

## Abbreviations

AUC: Area under the curve
EID–CT: Energy integrating detector CT
EUS: Endoscopic ultrasound
FNA: Fine–needle aspiration
HU: Hounsfield Unit
IPMN: Intraductal papillary mucinous neoplasm
MCC: Matthew’s correlation coefficient
MCN: Mucinous cystic neoplasm
NPV: Negative predictive value
PCD–CT: Photon–counting detector CT
PCL: Pancreas cystic lesion
PCN: Pancreas cystic neoplasm
PPV: Positive predictive value
ROI: Region of Interest
SCN: Serous cystic neoplasm
SPN: Solid pseudopapillary neoplasm
VNC: Virtual non–contrast
