# Supplementary material for: Spectral attenuation curves of photon-counting detector CT scans for the differentiation between mucinous and non-mucinous cyst fluids of pancreatic lesions – a proof-of-concept study
Source: BMC Med Imaging. 2025 Jul 1;25:239. doi: 10.1186/s12880-025-01770-6 (PMC12219141; doi:10.1186/s12880-025-01770-6)
Supplement: Supplementary file 1 — Supplementary Material 1 [file 12880_2025_1770_MOESM1_ESM.docx]

**Spectral attenuation curves of photon-counting detector CT scans for the differentiation between mucinous and non-mucinous cyst fluids of pancreatic lesions – a proof-of-concept study**

## Imaging protocol

The patients were examined at our institution with a PCD-CT scanner (NAEOTOM Alpha; Siemens Healthcare GmbH, Forchheim, Germany) according to our routine pancreas imaging protocol, which included an unenhanced scan, followed by arterial phase, pancreatic phase, and venous phase postcontrast scans. The patients were asked to drink at least half a liter of water before the examination as an intraluminal negative contrast medium. All patients were scanned in a supine position with both arms elevated. For the postcontrast phase series, a nonionic iodinated contrast agent (either Ultravist 370 or Iomeron 350) was injected at an injection rate of 2.4-4.3 mL using a power injector followed by a 40 mL saline chaser. The bolus tracking technique triggered by the peak contrast enhancement measured in the thoracic aorta was used for automated timing of the contrast injection. The timing for the arterial phase, pancreatic phase, and venous phase series were 23 s, 45 s, and 75 s, respectively. The amount of contrast agent was adjusted to the patient’s body weight.

The scans were performed with a tube voltage of 120 kVp and automated tube current modulation. The rotation time was 0.5 s, the pitch was 0.80, the single collimation width was 0.40 mm, the total collimation width was 57.60 mm, and the reconstruction matrix was 512x512 for all scans. Axial reconstructions of the CT series were generated with an SPP spectral reconstruction algorithm using a Qr40 soft kernel and a Q3 quantum iterative reconstruction algorithm with a slice thickness of 2.0 mm and an increment of 1.5 mm.
